# Health perception and restorative experience in the therapeutic landscape of urban wetland parks during the COVID-19 pandemic

**DOI:** 10.3389/fpubh.2023.1272347

**Published:** 2023-10-04

**Authors:** Jiang Li, Yating Chang, Xiaoxi Cai, Shaobo Liu, You Peng, Tao Feng, Jialing Qi, Yifeng Ji, Yiting Xia, Wenbo Lai

**Affiliations:** ^1^School of Architecture and Art, Central South University, Changsha, Hunan, China; ^2^School of Art and Design, Hunan First Normal University, Changsha, Hunan, China; ^3^Department of the Built Environment, Eindhoven University of Technology, Eindhoven, Netherlands; ^4^Graduate School of Advanced Science and Engineering, Hiroshima University, Hiroshima, Japan; ^5^School of Architecture, South China University of Technology, Guangzhou, Guangdong, China

**Keywords:** health perception, restorative experience, therapeutic landscape, COVID-19 pandemic, latent class analysis, structural equation modeling

## Abstract

**Introduction:**

The effects of restoration and inspiration in the therapeutic landscape of natural environments on visitors during the COVID-19 pandemic have been well-documented. However, less attention has been paid to the heterogeneity of visitor perceptions of health and the potential impacts of experiences in wetland parks with green and blue spaces on visitors’ overall perceived health. In this study, we investigate the impact of the restorative landscapes of wetland parks on visitors’ health perceptions in the context of the COVID-19 pandemic.

**Methods:**

In our survey, 582 respondents participated in an online questionnaire. We analyzed the respondents’ health perceptions in terms of latent class analysis, used multinomial logistic regression to determine the factors influencing the potential categorization of health perceptions, and used structural equation modeling to validate the relationships between health perceptions of different groups and landscape perceptions of wetland parks, restorative experiences, and personality optimistic tendencies.

**Results:**

The results identified three latent classes of health perceptions. Gender, marital status, education, occupation, income, distance, frequency of activities, and intensity of activities were significant predictors of potential classes of perceived health impacts among wetland park visitors.

**Discussion:**

This study revealed the nature and strength of the relationships between health perception and landscape perception, restorative experience, and dispositional optimism tendencies in wetland parks. These findings can be targeted not only to improve visitor health recovery but also to provide effective references and recommendations for wetland park design, planning, and management practices during and after an epidemic.

## 1. Introduction

The COVID-19 pandemic has had a profound impact on human society (1). Although control measures have calmed the outbreak, they have also exacerbated mental health problems (2, 3). A growing body of literature underlines the importance of extended psychological and physical health distress during the COVID-19 epidemic (4, 5), which is considered a public health concern. Although people may suffer from many adverse effects on their health during an epidemic, such as fear of viral infection, prolonged isolation, and other complex factors, people with optimistic dispositions tend to ease stress and anxiety caused by the epidemic because of their positive psychological cues, improve their physical health, seek social interactions to fulfill their social roles, and maintain their intrinsic mental and emotional health (6, 7).

Natural areas can provide an escape from mundane urban life (8). Many studies have suggested that natural connectivity in a therapeutic landscape provides a comfortable and healing environment for tourism and recreation (8–10). This reduction in mental and physical resources can be offset by access to a therapeutic environment (11, 12). Thus, visits to wetland parks, which are special parks with therapeutic landscapes in urban areas, may effectively prevent anxiety and depression caused by short- and long-term quarantines and lockdowns in natural environments (13–15). Wetland parks in urban areas improve water and air quality, support biodiversity, and promote human comfort (16, 17). However, less attention has been paid to the important role of therapeutic landscapes in urban wetland parks in healing, restoring, and maintaining human health (18–20). Therefore, it is paramount to understand how the experience of wetland parks benefits visitors’ perceived health. This study attempts to broaden the knowledge of how the experience of wetland parks’ therapeutic environment affects people’s health perceptions.

In addition, evidence shows differences in individuals’ perceptions of their health status based on gender, age, personality traits, and cultural background. These heterogeneities can directly influence visitors’ attitudes and behaviors toward health issues. Highly preferable and stimulating landscapes in wetland parks can improve people’s mental state and promote physical wellbeing. However, factors such as social background and personal preferences differ between individuals, leading to differences in their subjective perceptions of health. Therefore, understanding the heterogeneity of visitors’ health perceptions during their visit to wetland parks is important for improving their health status. Heterogeneity in health perceptions has become an important issue in several fields, including social psychology and behavioral medicine. However, academic research on health perception in wetland parks is lacking. Therefore, further research is needed to understand the heterogeneity in the health perceptions of wetland park visitors and guide the planning and management of wetland park landscapes.

The following section proposes a comprehensive framework through which we explore the nature and strength of the relationships between people’s perceived health and their experiences with the therapeutic landscape in terms of landscape perception, restorative experiences, and dispositional optimism tendencies in wetland parks. This is followed by a description of the methods used for data collection and modeling approaches, including latent class analysis (LCA) and structural equation modeling (SEM). Based on these results, the key findings and discussion are presented in Section 4. Finally, conclusions and implications are presented in the last section.

## 2. Conceptual framework and hypothesis

The landscape comprises physical, social, and symbolic aspects (21). People perceive the physical landscape as the interaction between individuals and the natural and built environments (22). The park, which is characterized by highly visible mowed areas, open water, and flourishing planting mixtures, provides a diverse and picturesque landscape for visitors to enjoy (23–27). It is attractive to walkers, dog walkers, and cyclists and provides space and opportunities for physical activity (28, 29). The social aspect of the landscape is perceived by individuals when they interact socially, and it includes a wide range of themes related to public attitudes, values, behaviors, and activities (30). The social aspect of the landscape benefits social integration by providing public spaces for activities that generate broad social connections and overcome people’s feelings of social isolation and exclusion (31, 32). The symbolic landscape, as a psychological cue, is associated with the public’s belief in the healing characteristics of the therapeutic environment (33–36). For example, national parks in the United States function prominently in American culture and religion, with symbolic meanings of ‘inspiration’, ‘stability’, and ‘healing’ (37).

By visiting urban wetland parks, people can access the therapeutic landscape of nature with water, diverse vegetation, open lawns, and bright flowers, resulting in substantial improvements in mood, cognitive function, and mental health (38–40). A temporary escape from the dreary pressures of work and life and immersion in nature can satisfy the need for fresh air, sunlight, water, and food, thus facilitating a restorative experience (41). Moreover, comfortable public spaces in wetland parks allow visitors to interact socially, independent of their socio-economic status (31, 32). Wetland parks provide uncrowded and undisturbed spaces for visitors seeking solitary experiences, such as meditation and independent thinking, in natural environments. In addition, visitors can achieve psychological benefits through horticultural activities that ultimately improve their perception of restoration (40).

According to the literature, an individual’s health is influenced by environmental factors, sociodemographic characteristics, and psychological traits (18, 39, 42, 43). Dispositional optimism is a positive psychological trait often associated with good health and relief from stress and anxiety (44). Therefore, experiences in wetland parks may have an impact on health perception, which may vary among individuals with various sociodemographic backgrounds and behavioral activities.

**Figure 1:**
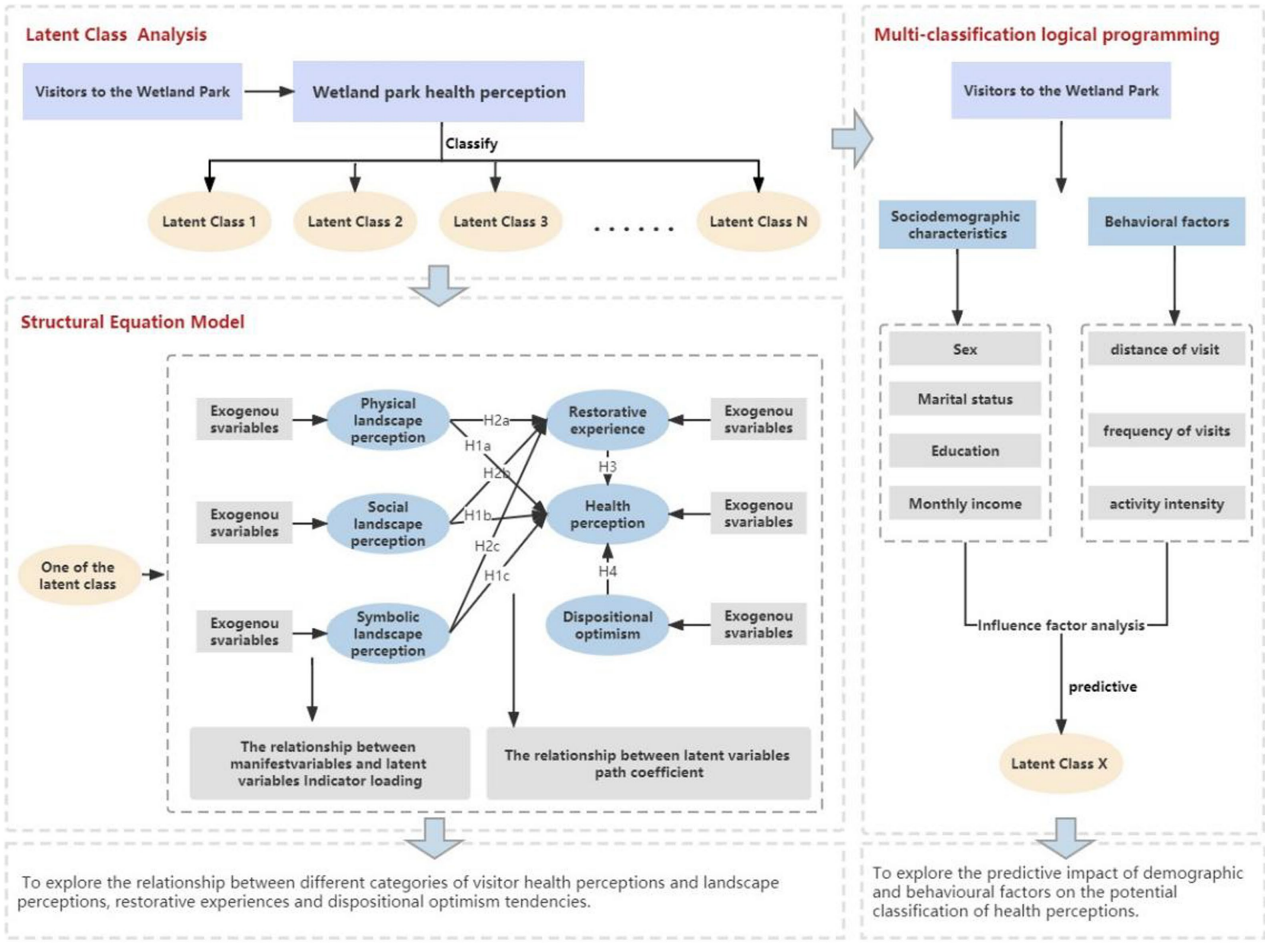
Modeling framework.

Previous studies have indicated a diversity in individuals’ health perceptions (45, 46). However, it cannot be simply determined *a priori* from the relevant observed variables because the unobserved heterogeneity in an individual’s perception is not necessarily captured by the variables preconceived and specified by existing theoretical and conceptual models, but it can exist outside of the previously identified variables (47). For example, the results of various studies on gender differences in health perceptions in wetland parks are mixed: Some studies have found no significant differences in health perceptions between men and women who visited wetland parks before and after the outbreak (39), while others have shown that women who visited nature reserves felt healthier than men (48). It is imperative to classify heterogeneous visitors into multiple homogeneous groups and identify appropriate treatments for wetland park planning and design. A new modeling framework (Figure 1) was developed to accurately capture the health behavior patterns and core characteristics of different populations.

## 3. Methodology

### 3.1. Methods

LCA is a finite mixture model. It is a statistical method that allows associations between exogenous indicators to be explained by an intermittent latent class so that associations between exogenous indicators are explained by the latent class and thus maintain their local independence (49, 50). LCA is used to identify unobservable classes within a population that may have similar health perceptions. It provides a medium through which results can be expressed in terms of probabilities rather than fixed deterministic conclusions (49, 51). The relationships between individuals’ landscape perceptions, restorative experiences, and dispositional optimism tendencies were analyzed separately for different classes of visitors.

The mathematical expression for LCA is shown in the following equation:


(1) $$\pi \begin{array}{c} \mathrm{AB}..ZX\\ \mathrm{ab}..zt \end{array}=\overset{T}{\underset{t}{\sum }}\pi \begin{array}{c} A|X\\ \mathrm{at} \end{array}\times \pi \begin{array}{c} B|X\\ bt \end{array}\times \cdots \times \pi \begin{array}{c} Z|X\\ zt \end{array}\times \pi \begin{array}{c} X\\ t \end{array}$$

where $\pi \begin{array}{c} \mathrm{AB}..ZX\\ \mathrm{ab}..zt \end{array}$ is the joint probability of a health-perceiving visitor taking the values of a,b..z for different field attributes. $\pi \begin{array}{c} A|X\\ \mathrm{at} \end{array}$ is a conditional probability indicating the conditional probability that the value of the health perception field attribute A of a health perception visitor is the given value of a given health perception visitor if the visitor belongs to class t of the latent variable. $\pi \begin{array}{c} x\\ t \end{array}$ is the latent class probability, which indicates the probability that a health-perceiving visitor belongs to potential class t.

The latent class probabilities are to satisfy equation (2).


(2) $$\underset{t}{\sum }\pi \begin{array}{c} x\\ t \end{array}=1$$

Conditional probability reflects the strength of the relationship between latent and exogenous variables. The formula used is as follows:


(3) $$\underset{a}{\sum }\pi \begin{array}{c} A|X\\ \mathrm{at} \end{array}=\underset{b}{\sum }\pi \begin{array}{c} B|X\\ bt \end{array}=\cdots \underset{z}{\sum }\pi \begin{array}{c} Z|X\\ zt \end{array}=1$$

SEM is a general statistical approach used to assess the relationships between observed and latent variables (52), and it can estimate specific path coefficients based on identified latent classes.

The equation of the measured model is as follows:


(4) X=Λxξ+δ

(5) Y=Λyη+ε

where equations (4) and (5) are the exogenous and endogenous indicators, respectively. Λ is the relationship between inventory and latent variables, and δ and ε are measurement errors.

The structural model is a path diagram reflecting the relationship between the effects of potential variables and is formulated as follows:


(6) η=Bη+Γξ+ς

where η is the endogenous latent variable, ξ is the exogenous latent variable, and Β denotes the effect of the exogenous latent variable on the endogenous latent variable. Γ denotes the effect of some endogenous latent variables on other endogenous latent variables, and ζ is the regression residual.

### 3.2. Data collection

The questionnaire consisted of seven sections: (I) socio-demographics, (II) physical landscape perception, (III) social landscape perception, (IV) symbolic landscape perception, (V) restorative landscape perception, (VI) health perception, and (VII) the C-WEMWBS Warwick-Edinburgh Positive Psychology Scale. The Health Perception Scale was derived from Ware’s General Health Perception Scale (53). The Restorative Experiences Scale was derived from Korpela (54), Korpela (55), and Von Lindern et al. (56). The Dispositional Optimism Scale was derived from the Warwick-Edinburgh Mental Health Scale (WEMWBS). Questionnaires were distributed across China *via* an online platform on 15 February 2022. Before the official questionnaire was distributed, a sample of wetland park visitors was taken for a pre-survey to verify its suitability and check for any ambiguities so that the questionnaire could be revised and improved. The survey was continued only if the respondents indicated their experience of visiting a wetland park. Table 1 shows the questionnaire form.

**Table 1:** Questionnaire scale form.

Latent variable	Code	Measuring question (Indicator)
Physical landscape perception (PLP)	PLP01	The wetland park has a large area of natural and human-made lakes and reservoirs
	PLP02	The wetland park is rich in animal and plant resources
	PLP03	The trees in the wetland park provide shade for pedestrians
	PLP04	The wetland park has enough open space and water-friendly leisure facilities
	PLP05	The wetland park has walkways for people
	PLP06	The wetland park provides a place to breathe fresh air
	PLP07	The wetland park provides a place to soak up the sun
	PLP08	The wetland park provides a place to observe nature and appreciate the beauty
Society landscape perception (SL)	SL01	The wetland park provides a relaxed social environment
	SL02	The wetland park provides a place for face-to-face communication
	SL03	In the wetland park, I can communicate and interact with my peers more actively
	SL04	I like to walk my dog or take my baby to the wetland park
	SL05	It is easier to find like-minded people with similar interests in wetland parks
	SL06	The wetland park promotes the development of restaurants and real estate around it
Symbolic landscape perception (SLP)	SLP01	The wetland park is rich in aquatic plants
	SLP02	Wetland landforms are rich in types
	SLP03	The wetland park can provide freshwater resources, aquatic products
	SLP04	Known as the ‘green lung of the city’, the park boasts a wide variety of plants
	SLP05	Wetland parks reduce urban carbon emissions
	SLP06	Wetland parks can mitigate runoff, store flood and prevent drought
	SLP07	Wetland parks have open water and are known as the ‘kidneys of the Earth’
	SLP08	Wetlands are called ‘cradles of species’
Restorative experience (PE)	PE01	In the wetland park, it can solve my mental health problems and psychological distress
	PE02	In the wetland park, I can experience harmony between humans and nature
	PE03	In the wetland park, I can get rid of the dull work pressure and life pressure temporarily
Health perception (HP)	HP01	My health is now excellent
	HP02	I do not think I’m ill
	HP03	I do not think I’m in sub-optimal health at the moment
	HP04	I feel better when I’m in the wetland park than when I’m not
	HP05	In the near future, I expect to have better health than other people I know
	HP06	I will not probably be sick a lot in the future
	HP07	I expect to have a very healthy life
	HP08	In the future, I will probably have fewer health problems than most people around me
	HP09	After visiting the wetland park, my sleep quality improved
	HP10	After visiting the wetland park, my breathing became more relaxed
	HP11	After visiting the wetland park, I had more energy
	HP12	After visiting the wetland park, I became more active
	HP13	After visiting the wetland park, my reflexes became stronger
	HP14	After visiting the wetland park, I look better
	HP15	After visiting the wetland park, my physical pain disappeared or lessened
Dispositional optimism (DO)	DO01	I always feel optimistic about the future
	DO02	I always thought I was useful
	DO03	I always feel relaxed
	DO04	I’m always willing to make new friends
	DO05	I’ve always been full of energy
	DO06	I have always been able to solve problems properly
	DO07	I’ve always been able to think clearly
	DO08	I’ve always been pleased with myself
	DO09	I’ve always felt connected to others
	DO10	I’ve always been confident
	DO11	I’ve always been able to make my own decisions
	DO12	I feel loved all the time
	DO13	I’m always in a good mood
	DO14	I’m always interested in new things

## 4. Results

### 4.1. Descriptive statistics

Table 2 presents the socio-demographic characteristics, socio-economic status, and dwelling conditions of the respondents. Slightly more female visitors than male visitors participated in the survey. Of the respondents, 18–45-year-olds were the majority, followed by those aged 45 years. In terms of marriage, 63.1% of the visitors were unmarried, and 36.9% were married. Approximately 76.1% of the respondents had a bachelor’s degree. Students were predominant in the sample population, accounting for 34.9% of respondents, while employed individuals accounted for 34.2%. The respondents’ monthly income is denoted in Chinese Yuan (CNY), and the income of more than one-third of the respondents did not exceed 3,000 CNY. Regarding housing tenure status, most respondents lived in dormitories or with their families, and approximately 70% of respondents did not own property. Most of the respondents had lived in the local area for more than 3 years. Nearly half of the respondents reported that their neighborhoods were under semi-containment or full containment as a result of the outbreak. Of these, 13.1% were strictly confined to their homes during the outbreak and could not move freely within their neighborhoods. In addition, 44% of them were required to record their temperature when entering and leaving the neighborhood.

**Table 2:** Background information of respondents (*n* = 582).

Variable	Count	Percentage
Gender		
Men	261	44.8%
Women	321	55.2%
Age (years)		
<18	13	2.2%
18–45	462	79.4%
>45	107	18.4%
Marital status		
Unmarried	367	63.1%
Married	215	36.9%
Education		
High school or less	85	14.6%
Senior or tertiary	54	9.3%
Bachelor’s degree	328	56.3%
Master and above	115	19.8%
Work status		
Students	203	34.9%
Unemployed or non-working	19	3.3%
Freelance	79	13.6%
Employed	199	34.2%
Employers	16	2.7%
Other	66	11.3%
Monthly income		
<3,000 CNY	198	34%
3,000–6,000 CNY	149	25.6%
6,000–9,000 CNY	103	17.7%
9,000–12,000 CNY	61	10.5%
12,000–15,000 CNY	35	6%
>15,000 CNY	36	6.2%
Housing		
Dormitory	203	34.9%
Apartment (rented and shared with other tenants)	52	8.9%
Apartment or studio (rented alone)	86	14.8%
Apartment (self-owned)	47	8.1%
Living with families	177	30.4%
Other	17	2.9%
Community management		
Fully locked	76	13.1%
Half-locked	256	44%
Open	250	42.9%
Local residential time		
<1 year	96	16.5%
1–3 years	154	26.5%
3–5 years	78	13.4%
>5 years	254	43.6%

Table 3 lists the factors related to transportation. More than half of the visitors traveled more than 5 km to the wetland park, and only 8.4% traveled less than 1 km; thus, the wetland park attracts many visitors who live far from it. In terms of transport choice, 41.6% of visitors chose to travel by taxi or car, with the fewest visitors choosing to travel by motorbike or bicycle, accounting for 11% of the sample size. The respondents who visited wetland parks once every 6 months accounted for 47.1% of the sample size, and only 2.6% visited wetland parks weekly. For most of them, the main purpose of visiting was to rest and relax. In terms of activity intensity, 84.2% of visitors chose low-intensity activities, whereas only 2.1% engaged in high-intensity activities. Regarding the form of accompaniment on a visit to a wetland park, 45.5% of visitors indicated that they usually visited a wetland park with friends, followed by family members, accounting for 39.7% of the sample size (Table 4).

**Table 3:** Traffic-related behavioral factors (*n* = 582).

Variable	Count	Percentage
The distance of most visited urban wetland park from residence		
< 1 km	49	8.4%
1–3 km	120	20.6%
3–5 km	107	18.4%
> 5 km	306	52.6%
Mode of transport		
Walking	79	13.6%
Bicycle or motorbike	64	11%
Public transport or underground	173	29.7%
By taxi or by car	242	41.6%
Other	24	4.1%

**Table 4:** Respondents’ behavioral factors (*n* = 582).

Variable	Count	Percentage
Frequency of visit		
Rarely go	77	13.2%
Half a year	274	47.1%
Once every 4–6 months a year	74	12.7%
Once every 2–3 months a year	67	11.5%
Once or twice a month	43	7.4%
Multiple times per month	32	5.5%
Every week	15	2.6%
Purpose		
Sports and fitness	64	11%
Rest and relaxation	405	69.6%
Social events	65	11.2%
Science activities	20	3.4%
Other	28	4.8%
Activity intensity		
Light strength	490	84.2%
Medium intensity	80	13.7%
High strength	12	2.1%
Companionship		
Alone	51	8.8%
Family members	231	39.7%
Friends	265	45.5%
Walking a dog	22	3.8%
Other	13	2.2%

### 4.2. Identifying distinct classes of health perception

Models with increments in the number of classes were estimated to detect a suitable number of latent classes. Multiple statistical indicators were used to determine the best-fit model. The smaller the values of the Akaike information criterion (AIC), Bayesian information criterion (BIC), and adjusted Bayesian information criterion (aBIC), the better the model’s fit (47, 57). Entropy evaluates the accuracy of the model classification, and a value closer to 1 indicates a better fit (58). The Lo–Mendell–Rubin (LMR) and bootstrapped likelihood ratio test (BLRT) provide a value of p that can be used to compare the increase in model fit between neighboring class models and to determine a statistically significant improvement in fit when another class is included. As shown in Table 5, the AIC, BIC, and aBIC decreased as the number of categories increased. It is possible that the higher the number of categories, the better the fit. However, from Class 3, the entropy started to decrease, and from Class 4, the LMR (P) also started to become statistically insignificant (*p* > 0.05), indicating that the model’s fit gradually deteriorated from Class 3. The entropy was closest to 1 in Class 3, which means that the model has the best-fit and classification accuracy. A model with three latent classes was selected as the most appropriate for this study, considering the practical significance of the classes and the fit values p represented by the classes.

**Table 5:** Estimated indices for comparing different numbers of latent classes.

Class number	AIC	BIC	aBIC	Entropy	LMP	BLRT
1	10261.499	10326.996	10279.376			
2	7069.446	7204.807	7106.393	0.954	0	0
3	6233.476	6438.7	6289.493	0.966	0	0
4	5939.374	6214.462	6014.461	0.931	0	0
5	5865.218	6210.169	5959.374	0.918	0.1261	0.1285

**Figure 2:**
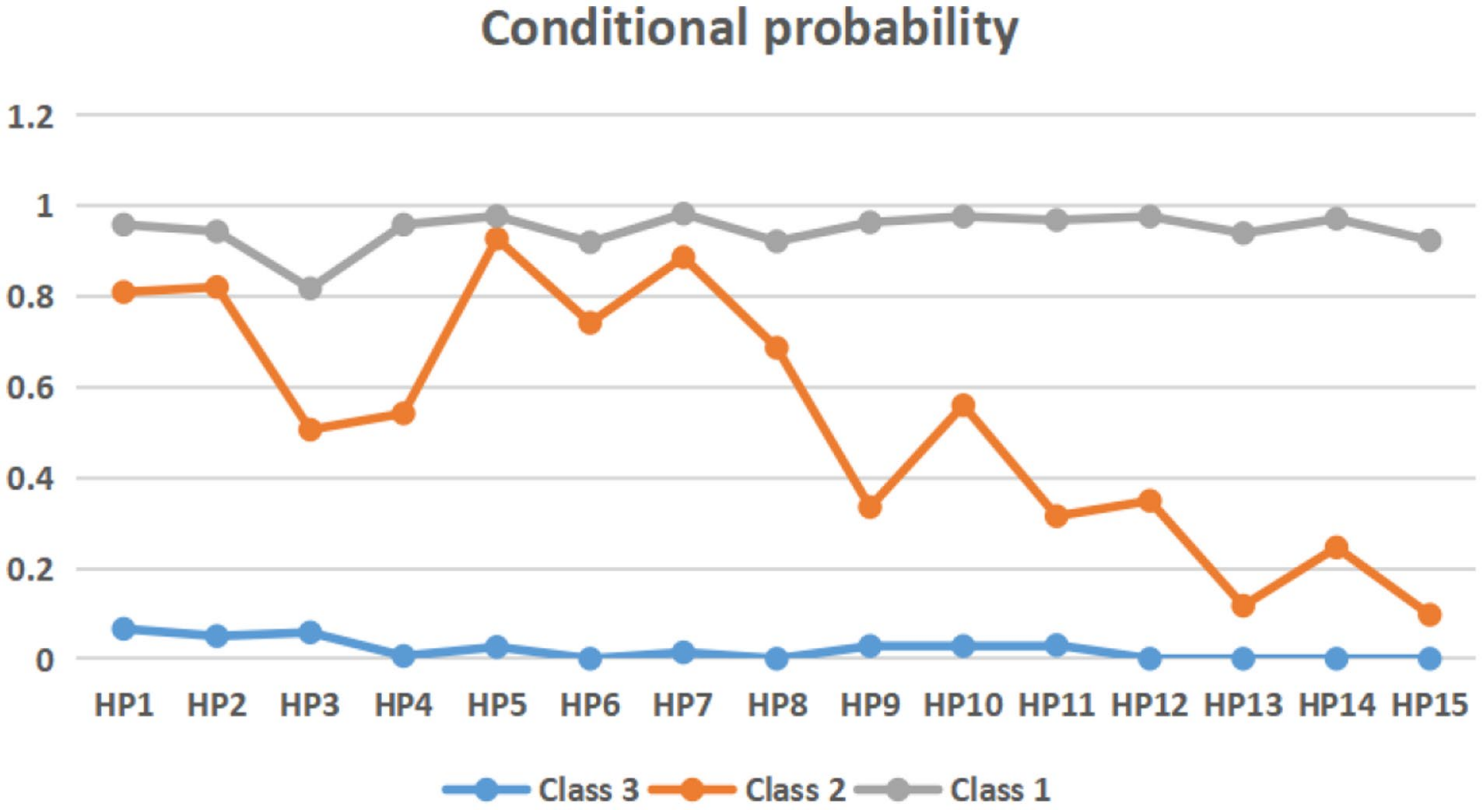
Conditional probability of three-class model.

Based on the three-class model, Figure 2 illustrates the probabilities of respondents’ health perception in each latent class. Latent class 1 (C1) was characterized by high probabilities for all health perception components and labeled ‘good self-perception of health’ (*n* = 323, 55.5%). Latent class 2 (C2) was characterized by high probabilities of good health status in the present and future but did not improve significantly after the visit to the wetland park and was, therefore, named ‘neutral self-perception of health’ (*n* = 192, 33.0%). Latent class 3 (C3) was characterized by low probabilities of all health perceptions; accordingly, we labeled it ‘poor self-perception of health’ (*n* = 67, 11.5%).

### 4.3. Roles of different factors in predicting class membership

We further conducted a multinomial logistic regression model to examine the respondents’ health perceptions associated with their social backgrounds and behavioral activities, with Class 2 (medium self-perception of health) as the reference group (Table 6).

**Table 6:** Results of multinomial logistic regression.

Independent variable	C1		C3	
	*P*	OR	*P*	OR
Gender (ref: men)				
Women	0.114	1.21	0.009	1.00
Marital status				
Unmarried	0.001	1.24	0	1.00
Education				
High school or technical secondary school	0.068	1.41	0.157	1.00
Higher vocational or junior college	0.136	1.48	0.022	1.07
Bachelor’s degree	0.695	1.21	0.361	1.15
Master and above	0.001	1.25	0.033	2.00
Work status				
Students	0.001	1.22	0.003	1.00
Freelance	0.008	1.36	0.108	1.00
Employed, Employers	0.442	1.22	0.96	1.01
Housing				
Group accommodation	0.015	1.22	0.027	1.00
Rent alone, share house	0.057	1.25	0.986	1.02
Living with family	0.344	1.24	0.071	1.06
Community Management				
Half-locked	0.035	1.21	0.002	1.00
Open	0.035	1.21	0.131	1.04
Distance from residence				
1–3 km	0.734	1.26	0.802	1.00
3–5 km	0.61	1.28	0.617	2.08
>5 km	0.79	1.21	0.028	1.84
Purpose				
Rest and relaxation	0.004	1.25	0	1.00
Social events	0.211	1.37	0.459	1.00
Science activities	0.305	1.85	0.247	1.23
Forms of companionship				
Family members	0.304	1.22	0.106	1.00
Friends	0.02	1.21	0.002	1.36
Walking a dog	0.308	1.70	0.722	1.02
Frequency of park visits before the epidemic				
Half a year	0.768	1.21	0.036	1.00
Once every 4–6 months a year	0.076	1.31	0.607	2.16
Once every 2–3 months a year	0.629	1.36	0.393	1.08
Once or twice a month	0.609	1.45	0.327	1.88
Multiple times per month	0.74	1.60	0.143	1.84
Every week	0	1.86	0.397	2.10

OR, dominance ratio; if the dominance ratio is greater than one, the probability of membership being classified into a particular class increases with each unit of the predictor variable. If the dominance ratio is less than 1, the probability of membership being classified into a particular class decreases for each unit of the predictor variable.

Compared with Class 2, respondents in Class 1 (good self-perception of health) were more likely to be unmarried (OR = 1.24, *p* < 0.001), master and above (OR = 1.25, *p* < 0.001), students or freelancers (OR = 1.22, *p* < 0.001; OR = 1.36, *p* < 0.01), in a group accommodation (OR = 1.22, *p* < 0.05) and under open and semi-closure management of residential areas during the outbreak period (OR = 1.21, *p* < 0.05; OR = 1.21, *p* < 0.05). The main purposes for visiting the park were more likely to be for rest and relaxation (OR = 1.25, *p* < 0.01). The probabilities of visiting the park with friends were high, and weekly park visits before the outbreak were high (OR = 1.21, *p* < 0.05; OR = 1.86, *p* < 0.001).

Compared with Class 2, respondents in Class 3 (poor self-perception of health) were more likely to be female (OR = 1, *p* < 0.05), unmarried (OR = 1, *p* < 0.001), with a higher vocational or junior college degree (OR = 1.07, *p* < 0.05), with a master’s degree and above (OR = 2, *p* < 0.05), students (OR = 1, *p* < 0.05), in a group accommodation (OR = 1, *p* < 0.05), and under semi-closure management of residential areas during the outbreak period (OR = 1, *p* < 0.01). Accommodation was more than 5 km away from the wetland park (OR = 1.84, *p* < 0.05). The main purposes of visiting the park were more likely to be for rest and relaxation (OR = 1, *p* < 0.001). The probabilities of visiting the park with friends and visiting the park 6 months before the outbreak (OR = 1.36, *p* < 0.05; OR = 1, *p* < 0.05).

### 4.4. Structural equation modeling results

Table 7 shows that the critical fitting indices of C1 and C2 are within the acceptable recommended value range; thus, models C1 and C2 have good fitting degrees. The CFI and TLI of C3 are within the acceptable recommended value range, respectively; however, the RMSEA of C3 suggests an insufficient model fit. Overall, the model’s fit was considered acceptable, as RMSEA can suggest a below-adequate model fit for models with low degrees of freedom (59). The relevant structural equation modeling adjustments are detailed in Appendix A.

**Table 7:** Structural equation model fit index values.

Accessory indicators	Fit		
	C1	C2	C3
X^2^	1715.787	1198.198	26.723
X^2^/df	2.186	1.517	1.782
RMSEA	0.061	0.052	0.109
CFI	0.912	0.911	0.971
TLI	0.903	0.903	0.945

**Figure 3:**
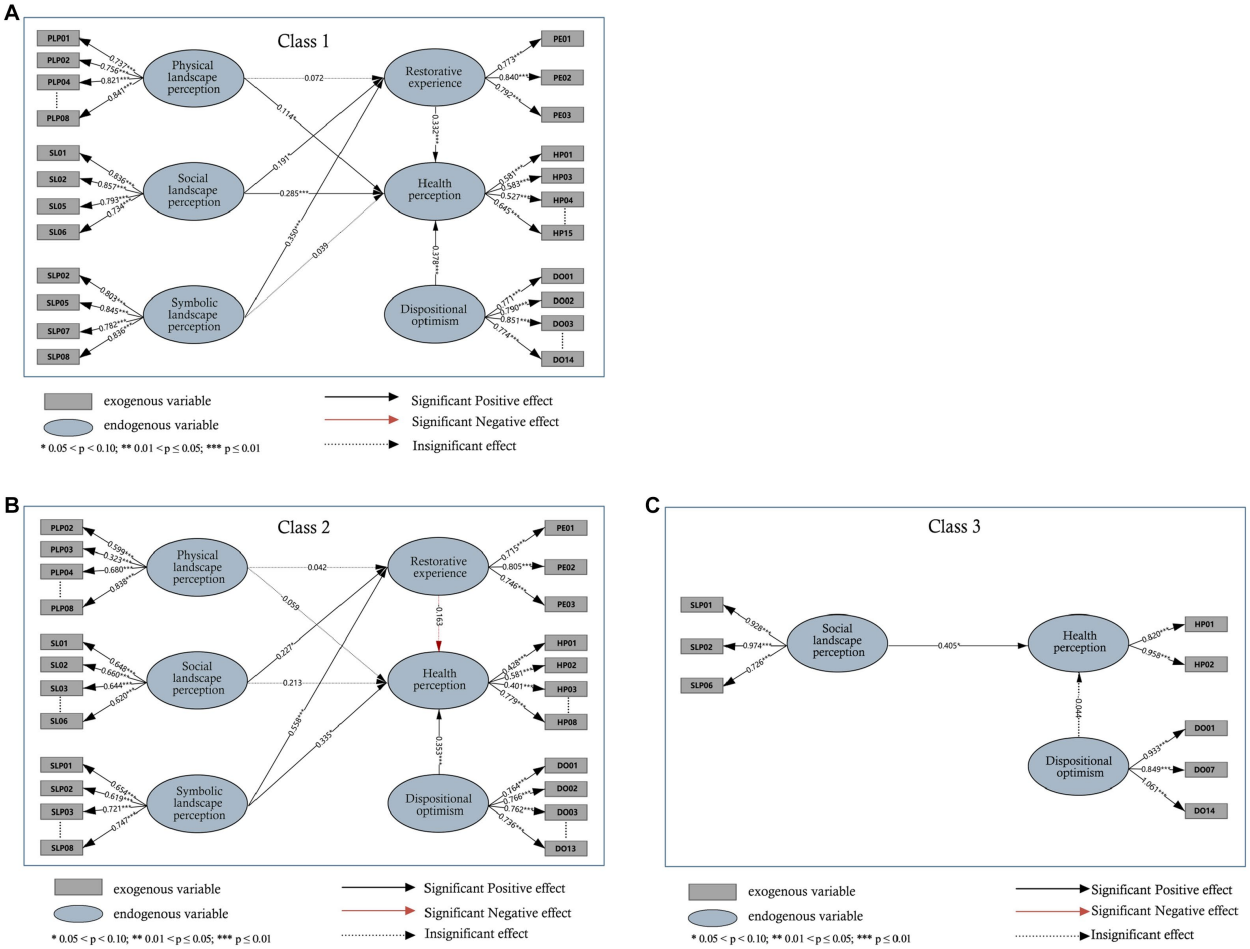
SEM estimation results: **(A)** Class 1. **(B)** Class 2. **(C)** Class 3.

The research model’s hypothesis was validated using AMOS 24.0, and the standardized path coefficients and significance levels between the latent variables are shown in Figure 3. The final hypothesis validation results are shown in Figure 3.

As shown in Tables 8–10, the standardized factor loadings between the latent variables and all the corresponding observed variables are >0.5. Regarding the structural models, Table 11 presents the estimation results for the three latent classes; those for latent class 1 are as follows (Figure 3A). Visitor’s physical landscape perception (*γ* = 0.114, *p* = 0.021) and social landscape perception (*γ* = 0.285*, p* <0.001) were positively significant on health perception. Visitors’ perceptions of the social landscape (*γ* = 0.191, *p* = 0.005) and symbolic landscape (*γ* = 0.350, *p* < 0.001) were positively significant for their restorative experiences. The effect of visitors’ restorative experience (*γ* = 0.332, *p*<0.001) and character optimism (*γ* = 0.378, *p*<0.001) on health perceptions was positively significant. The relationship between the visitors’ symbolic landscape perception variables (*γ* = 0.039, *p* = 0.526) and health perceptions was not significant.

**Table 8:** Results of the measurement model of C1.

Variables	Estimate	*p*	Variables	Estimate	*p*
Physical landscape perception			Health Perception		
PLP01	0.737	***	HP01	0.581	***
PLP02	0.756	***	HP03	0.527	***
PLP04	0.821	***	HP04	0.598	***
PLP05	0.860	***	HP08	0.583	***
PLP06	0.908	***	HP09	0.726	***
PLP07	0.845	***	HP10	0.748	***
PLP08	0.841	***	HP11	0.691	***
Social landscape perception			HP12	0.726	***
SL01	0.836	***	HP13	0.666	***
SL02	0.857	***	HP14	0.702	***
SL05	0.793	***	HP15	0.645	***
SL06	0.734	***	Dispositional optimism		
Symbolic landscape perception			DO01	0.771	***
SLP02	0.803	***	DO02	0.790	***
SLP05	0.845	***	DO03	0.851	***
SLP07	0.782	***	DO04	0.806	***
SLP08	0.836	***	DO05	0.851	***
Restorative experience			DO06	0.845	***
PE01	0.773	***	DO07	0.857	***
PE02	0.840	***	DO08	0.914	***
PE03	0.792	***	DO10	0.855	***
			DO11	0.831	***
			DO12	0.781	***
			DO13	0.856	***
			DO14	0.774	***

***p < 0.01, **0.01 ≤ *p* < 0.05, *0.01 ≤ *p* < 0.1.

**Table 9:** Results of the measurement model of C2.

Variables	Estimate	*p*	Variables	Estimate	*p*
Physical landscape perception			Restorative experience		
PLP02	0.599	***	PE01	0.715	***
PLP03	0.623	***	PE02	0.805	***
PLP04	0.680	***	PE03	0.746	***
PLP05	0.831	***	Health perception		
PLP06	0.811	***	HP02	0.581	***
PLP07	0.756	***	HP03	0.401	***
PLP08	0.838	***	HP06	0.675	***
Social landscape perception			HP07	0.543	***
SL01	0.648	***	HP08	0.779	***
SL02	0.660	***	Dispositional optimism		
SL03	0.644	***	DO01	0.764	***
SL04	0.668	***	DO02	0.766	***
SL05	0.737	***	DO03	0.762	***
SL06	0.620	***	DO04	0.749	***
Symbolic landscape perception			DO05	0.794	***
SLP01	0.654	***	DO06	0.839	***
SLP02	0.619	***	DO07	0.789	***
SLP03	0.721	***	DO08	0.771	***
SLP04	0.686	***	DO09	0.752	***
SLP05	0.664	***	DO10	0.850	***
SLP06	0.776	***	DO11	0.669	***
SLP07	0.757	***	DO13	0.736	***
SLP08	0.747	***			

***p < 0.01, ** 0.01 ≤ *p* < 0.05, *0.01 ≤ *p* < 0.1.

**Table 10:** C3: Results of the measurement model.

Variables	Estimate	*p*
Social landscape perception		
SL01	0.928	***
SL02	0.974	***
SL06	0.726	***
Health perception		
HP01	0.820	***
HP02	0.958	***
Dispositional optimism		
DO01	0.933	***
DO07	0.849	***
DO14	1.061	***

**Table 11:** Results of the structural model.

hypothesis	C1			C2			C3		
	Estimate	*p*-Value	Conclusion	Estimate	*p*-Value	Conclusion	Estimate	*p*-Value	Conclusion
H1a	0.114	0.021	Support	−0.059	0.636	Not supported	\	\	\
H1b	0.285	***	Support	0.213	0.067	Not supported	0.405	0.015	Support
H1c	0.039	0.526	Not supported	0.335	0.035	Support	\	\	\
H2a	0.124	0.072	Not supported	0.042	0.694	Not supported	\	\	\
H2b	0.191	0.005	Support	0.227	0.011	Support	\	\	\
H2c	0.350	***	Support	0.558	***	Support	\	\	\
H3	0.332	***	Support	−0.163	0.239	Not supported	\	\	\
H4	0.378	***	Support	0.353	***	Support	−0.044	0.725	Not supported

*** indicates *p* < 0.001, ** indicates *p* < 0.01, * indicates *p* < 0.05.

The results in C2 were as described below (Figure 3B). In terms of perceived landscape, perceived symbolic landscape (*γ* = 0.335, *p* = 0.035) had a positive and significant effect on health perception. Restorative experiences (*γ* = −0.163, *p* = 0.239) and health perceptions were not significantly related to each other. Dispositional optimism (*γ* = 0.353, *p*<0.001) significantly and positively influenced health perceptions. In terms of restorative experience, the perceived physical landscape (*γ* = 0.042, *p* = 0.694) was not significantly related to restorative experience. Perceived social landscape (*γ* = 0.227, *p* = 0.0.011) and symbolic landscape (*γ* = 0.558, *p* < 0.001) significantly influenced restorative experience.

For latent class 3 (Figure 3C), social landscape perception (*γ* = 0.405, *p* = 0.015) influenced health perception, while dispositional optimism (*γ* = −0.044, *p* = 0.725) had no significant relationship with health perception.

## 5. Discussion

We investigated the impact of restorative landscapes in wetland parks on the health perceptions of respondents during the COVID-19 pandemic. By classifying respondents’ health perceptions into latent classes, we found that visitors could be classified into three groups with significant group heterogeneity in the population. Sociodemographic characteristics and behavioral factors showed different associations with each group.

Compared with the ‘medium self-perception of health’ class, respondents in the ‘good self-perception of health’ group were more likely to be freelancers and have a higher education level. Consistent with previous studies, freelancers have more time at their disposal and fewer stressors than other professionals, and participating in leisure and recreational activities can reduce psychological stress and improve their physical and mental wellbeing. With the improvement in education level, highly educated people have a relatively high preference for wild nature and a strong restorative experience in wetland parks (23). Our results also showed that residents of unsealed neighborhoods had a higher probability of belonging to the ‘good self-perception of health’ category during the peak of the epidemic. Lockdown measures have been a panacea for pandemic control. However, home restrictions and the overall disruption of personal daily life have made people exercise less; the workspace in a home office environment has increased the chances of physical pain and other physical health conditions, and blurred work–life boundaries can make it difficult to detach mentally from work, which can increase stress and anxiety. Therefore, when dealing with major safety and health events such as the epidemic, the community and the wetland park should adopt a ‘closed’ and ‘open’ relationship (12, 60–64). The frequency and intensity of park cleaning and disinfection can be increased within the park, and infrastructure to stimulate exercise can be incorporated. The maximum number of visitors to a park during an outbreak can also be predicted, and spaces with a large distribution of visitors can be monitored for flow and effective evacuation guidance. Outside the parks, it is necessary to increase connections with residents in neighboring communities and improve residents’ accessibility to parks (65).

Compared to the ‘medium self-perception of health’ group, the ‘poor self-perception of health’ group was more likely to include women. Consistent with previous studies, gender strongly shapes the experience of visitors to urban parks, with men being more likely than women to rate health benefits when visiting urban blue-green spaces at peak times. In addition, the more frequently residents visit parks, the better their perceived health status, which indicates that park users develop place attachment and increased affinity with nature, which contribute to health benefits. The more residents lived in a wetland park, the worse their perceived health. This is because, at the peak of the epidemic, many non-essential commercial and public spaces were off-limits. People’s need to visit outdoor spaces was higher than ever. Thus, the closer the urban wetland park was to the neighbourhood, the more people had access to the park, and the more likely they felt healthy. Therefore, it is possible to extend the opening hours of wetland park services during epidemics and improve accessibility to parks while observing more accurate infection prevention methods to ensure wetland park use (65, 66).

The SEM results of the three categories showed that landscape perception, optimistic personality tendencies, and restorative experiences had different strengths and associations with health perception. It is worth noting that in the first category, the symbolic landscape has no positive effect on health perception and restorative experience, possibly because the environmental elements and cultural cues associated with symbolic restorative health in China’s wetland parks have not yet established positive images for people. Fewer psychological hints about health are available to visitors compared to those available at famous sites known for longevity and healing. Therefore, in future construction of wetland parks, planning designers can try to add cultural clues and symbols with symbolic significance for health and create environmental factors conducive to the healthy restoration of wetland parks. Additionally, the results showed that optimism had an impact on health perceptions regardless of group, and optimists’ positive expectations are not limited to specific areas of behavior or types of circumstances.

## 6. Conclusion

This study examined the impact of wetland park landscapes on different health-perceiving populations during the COVID-19 pandemic. The study identified various factors affecting the potential categories of health perception of visitors, such as gender, marital status, education, work status, housing, community management, distance from home, purpose, form of companionship, and frequency of pre-pandemic park visits. The existence of different pathways and correspondence coefficients among the three potential categories of visitor groups was also investigated. In the event of a future major safety and health event, wetland park managers should pre-emptively address the need for ‘closed’ and ‘open’ relationships between communities and wetland parks and try to increase cultural cues and symbols with health symbolism. However, this study had several limitations. First, the findings were limited to Chinese participants. Future studies should explore cross-cultural comparisons among visitors from different countries. Second, this study did not examine the dynamics of health perceptions over time. Future research could examine longitudinal changes in the health perceptions of wetland parks. These insights can inform the decision-making process and contribute to better planning and management of wetland parks.

## Data availability statement

The original contributions presented in the study are included in the article/supplementary material, further inquiries can be directed to the corresponding authors.

## Ethics statement

Ethical review and approval was not required for the study on human participants in accordance with the local legislation and institutional requirements. Written informed consent from the participants was not required to participate in this study in accordance with the national legislation and the institutional requirements.

## Author contributions

JL: Conceptualization, Funding acquisition, Resources, Supervision, Writing – original draft. YC: Writing – original draft, Data curation, Formal analysis, Investigation, Methodology. XC: Conceptualization, Funding acquisition, Resources, Writing – review & editing. SL: Funding acquisition, Resources, Supervision, Writing – review & editing. YP: Conceptualization, Methodology, Writing – review & editing. TF: Writing – review & editing. JQ: Formal analysis, Investigation, Writing – original draft. YJ: Formal analysis, Investigation, Writing – original draft. YX: Formal analysis, Investigation, Writing – original draft. WL: Resources, Writing – review & editing.

## Appendix A

**Table:**

Number	Step
1	Model construction: constructing a hypothetical model based on theory requires making assumptions about the relationship between observed variables and latent variables, the interrelationships between each latent variable, etc.
2	Model fitting: The main purpose is to use the sample to estimate the model parameters and to find the set of parameters that minimize the “gap” between the implied covariance matrix of the model and the sample covariance matrix.
3	Model evaluation: significance of path coefficients, reasonableness of the relationship of each parameter to the pre-determined model, passing of each fitting index.
4	Model correction: When the evaluation indicators are not satisfactory, the model is corrected, either by model expansion (using correction indices) or by model restriction (using critical ratios).
	MI amendment: Use the modification index to make corrections to the model.
	Reduction of measurement indicators:Remove terms that do not make sense to ensure that the final measurement model is good. Specifically, exploratory factor analysis was conducted upfront, followed by the use of validation factor analysis, and structural equation modeling was carried out after the measurement relationship was indeed fine, to reduce the interference that a ‘bad measurement relationship’ would bring to the model.
	Path reduction: Combining explicit and tacit knowledge from the domain and context, impact pathways that did not reach the significance level were removed,
5	modeling summary
